# Fiber-optic endoscopic evaluation of swallowing to assess swallowing outcomes as a function of head position in a normal population

**DOI:** 10.1186/1916-0216-43-9

**Published:** 2014-04-22

**Authors:** Lucas A Badenduck, T Wayne Matthews, Alanna McDonough, Joseph C Dort, Kristin Wiens, Rachelle Kettner, Susan Crawford, Bonnie J Kaplan

**Affiliations:** 1 Faculty of Medicine, University of Calgary, Calgary, Alberta, Canada; 2 Foothills Medical Centre, Calgary, Alberta, Canada; 3 Alberta Children’s Hospital, Calgary, Alberta, Canada; 4 Department of Surgery, University of Calgary, Suite 602 South Tower, Foothills Medical Centre, 1403 29th St. NW, Calgary, Alberta T2N T29, Canada

**Keywords:** Deglutition, Dysphagia, FEES, Penetration, Aspiration

## Abstract

**Background:**

Head position practice has been shown to influence pill-swallowing ability, but the impact of head position on measures of swallowing outcomes has not yet been studied with fiber-optic endoscopic evaluation of swallowing (FEES). The primary purpose of this study was to determine whether head position impacts penetration-aspiration scale scores and/or post-swallow pharyngeal residue as assessed by FEES. Documenting the incidence of pharyngeal residue and laryngeal penetration and aspiration in a normal population was a secondary goal.

**Methods:**

Adults without swallowing difficulties (N = 84) were taught a pill swallowing technique based on learning five head positions and were asked to practice with small, hard candies (e.g., TicTacs) for two weeks. Then they demonstrated swallowing in each of the head positions for two conditions, liquid and purée, while undergoing FEES.

**Results:**

Out of 840 examined swallows, one event of aspiration and 5 events of penetration occurred. During practice >50% participants found positions they preferred over the center position for swallowing but head position was not associated with penetration-aspiration scores assessed by FEES. Significant associations and non-significant trends were found between pharyngeal residue and three variables: age, most preferred head position, and least preferred head position.

**Conclusion:**

Head position during swallowing (head up) and age greater than 40 years may result in increased pharyngeal residue but not laryngeal penetration or aspiration.

## Level of evidence: 4 (case series)

Fiber-optic Endoscopic Evaluation of Swallowing (FEES) is commonly employed in the office by otolaryngologist – head and neck surgeons and speech language pathologists, and yet a description of office-based FEES findings in a normal population has not been described. Previous research has found that head position can influence the process of swallowing. Ohmae reported that lateral rotation of the head (left or right) significantly decreased upper esophageal sphincter (UES) pressure while also increasing the length of time that the UES remains open [1]. In addition, they found that the bolus was directed towards the opposite side of the head rotation. A study by Logemann et al. [2] showed that the UES opening diameter increased with lateral head rotation. Other research has also found that the chin down, otherwise known as the chin tuck, head position can have a positive effect on swallowing with patients after head and neck surgery [3]. Physical changes, such as the narrowing of the airway entrance, are associated with the head flexed position and can aid in the protection of the airway during swallowing [4]. Head extension has been found to decrease UES relaxation time, increase UES residual pressure and narrow the pharyngoesophageal junction [5].

Based on the knowledge that head position can alter swallowing dynamics, Kaplan and colleagues investigated the effect of different head positions on ease of pill swallowing [6]. The behavioral technique they eventually developed involves the teaching and practicing of five different head positions for swallowing: center, chin tilted up, chin tilted down, head rotated left and head rotated right. The various head positions were explored because, for instance, it was thought that the decreased UES pressure and extended opening time reported by Ohmae et al. [1] and Logemann et al. [2] might provide for easier passage of pills into the esophagus.

The first study by Kaplan et al. [6] involved 106 male and female participants aged 8–40 years with no pill swallowing difficulties. The participants were taught the five head positions and asked to report on how each of the head positions felt. Because the majority of those individuals reported discomfort in positions never previously attempted, all subsequent studies (including the one reported herein) required two weeks of daily practice in all positions to promote habituation to the new sensations. Generally, the practice session data have not been collected or analyzed. The second study investigated the effect of daily practice of the different head positions for a two-week period of time in 134 male and female adults with no identified pill swallowing difficulties. These adults, aged 18–30 years, were taught the five different head positions; they practiced for two weeks while rating each position in terms of comfort. After incorporating the process of two weeks of practice, the third study involved 108 male and female participants aged 18–30 with self-reported swallowing difficulties.

The protocol that was developed from working with >300 participants in these three studies was then applied to 33 clinically referred children who had lifetime histories of being unable to swallow pill-form medications. Each child was able to learn how to swallow pills successfully within two weeks. Importantly, the total time for the intervention was only about two hours (initial training meeting of 45 minutes, the 5 minutes of practice a day for two weeks and a final meeting of 10 minutes), in comparison to previous interventions using classical behavioral techniques (e.g., stimulus shaping and reinforcement), some which required as much as 10 hours [7] or others that required less time but also were less successful [8,9]. As a result of these four studies, an educational video was created (http://www.research4kids.ucalgary.ca/pillswallowing).

The successful application of head position training to this clinical problem led to the question of whether head position was associated with differences in swallowing function in normal subjects that would be detectable with physiological investigations. FEES was selected as the method of objectively investigating important swallowing outcomes. The procedure allows direct visualization of pharyngeal residue and laryngeal penetration of foods of different consistencies immediately after swallowing. This office procedure, commonly used by otolaryngologist – head and neck surgeons and speech language pathologists, compares favorably to modified barium swallow in the ability to detect pharyngeal residue and laryngeal penetration and aspiration [10]. The clinician investigators perform FEES without requiring access to imaging department resources. Exploring the relationship between head position and swallowing function assessed by FEES in a population of normal adults is the primary objective of the current study. Our secondary objective is to describe the incidence of pharyngeal residue, penetration and aspiration in this population overall and when stratified by age, gender and preferred head position.

## Methods

### Participants

The study cohort consisted of males and females aged 18 and over that did not have difficulty swallowing pills. Subjects were recruited using flyers, advertisements, and word of mouth as well as through a University undergraduate program in Psychology where students received a small amount of bonus credit for participating in approved research. Eighty-five adults volunteered, but one participant withdrew partway through the FEES procedure. Although she was not choking, the scope appeared to trigger a gag reflex that made it impossible for her to continue.

### Procedure

When a volunteer subject responded to an advertisement, a research assistant arranged a first appointment, at which the study was explained, consent forms were signed, the five head positions (head centre, chin up, chin down, rotated left and right) were demonstrated and practiced, and a supply of small hard candies was provided. The volunteer was asked to practice each of the five positions with the candies once/day for 14 days prior to a FEES appointment. They were also asked to complete a daily report on a record sheet. As mentioned, the purpose of practice was to habituate to swallowing in all head positions prior to the FEES appointment, so that when the flexible laryngoscope was inserted, performing the task of swallowing in various head positions would be sufficiently automatic that the FEES procedure could be completed quickly.

After at least 14 days of practice, the volunteer subject was scheduled for a FEES appointment. When greeted at that appointment, the practice sheets were collected to ensure that they were compliant with the study expectations, and the FEES methodology was explained. All participants returned completed practice sheets (though the actual practice information was not analyzed). All FEES procedures were done using a Karl Storz flexible fiber-optic rhinolaryngoscope (Karl Storz Endoscopy, Tuttlingen, Germany) without the application of topical anaesthesia. The video capture system consisted of an analog camera (Karl Storz) connected to a Medicapture USB 200 digital image recorder (Medicapture, Plymouth Meeting, PA, USA). The images were digitized by the imaging system. All video sessions were recorded and saved in mpeg2 format on an external hard drive allowing for post hoc review by the investigators.

The fiberoptic endoscope, lubricated with a non-anaesthetic gel, was passed trans-nasally until the larynx and hypopharynx were visualized. The examining otolaryngologist usually began with the right nostril, but if endoscope passage was difficult, the left nostril was used. The nostril selected for the endoscope was recorded for each participant.

Once the endoscope was in place, participants were taken through ten swallows. Each of the five head positions had two distinct swallow conditions using two textures: 5-mL thin liquid (milk) and 5-mL purée (applesauce). Both the milk and applesauce were dyed with green food coloring to aid in visualization and were delivered with a 5-mL teaspoon. Participants were asked to hold the liquid or purée in their mouth until instructed to swallow. When participants were instructed to swallow, during each condition, they were told to swallow as if they were swallowing a pill. During the swallow, the tip of the endoscope was positioned in the posterior nasopharynx. Immediately after the swallow, the endoscope was advanced forward into the oropharynx and supraglottic larynx to assess penetration and aspiration, and residue. Other than the single subject who withdrew from the study, there were no adverse events associated with the FEES procedure.

### Measures

Penetration-Aspiration was evaluated by consensus between two individuals, an otolaryngologist (either TWM or JCD) and a speech-language pathologist (AM), for each swallow, using the 8-point Penetration-Aspiration Scale developed by Rosenbek et al. [11]. Post-swallow pharyngeal residue was scored in the same way, using an adapted 4-point residue scale developed by Butler and colleagues [12]. Thus, each swallow was assigned a score from 1 to 8 for penetration/aspiration, as well as a score from 0 to 3 for residue. After completing the FEES procedure, when the information could not bias the scores, participants were asked which head position (or positions) had been the most- and least-preferred during the 14 days of practice with candies.

### Analysis

Data were analyzed using SPSS 15.0.1. All analyses that included most (or least) preferred head positions utilized the first-order Rao-Scott corrected chi-square, which allowed us to account for multiple participant responses [13]. In total, 12 participants did select multiple options for their most preferred head position and 2 selected multiple options for their least preferred position, all of which required the Rao-Scott correction. Other analyses used the chi square test. For residue, scores were categorized as per Butler et al. [12], with scores of 0 and 1 (defined as normal) as well as 2 and 3 (defined as abnormal). Statistical significance was set at p < .05.

## Results

### Participants

In total, there were 84 participants aged 18 – 72 years (32 males, 52 females; average age 32.2 years, SD = 15.7). The majority (82.1%) had the endoscope passed through their right nostril. No one reported having difficulties swallowing. Fifty-two were in the pre-defined ‘younger’ age group` of 18–39, and 27 in the ‘older’ age group (40 years and above).

The reports on most- and least-preferred head positions after two weeks of practice with candies revealed that many participants maintained preference for swallowing with the head in a neutral horizontal plane (70.3% (N = 59)), (with a head center being the most commonly preferred position (N = 29 (34.5%)). (see Table 1). Head positions above or below the horizontal plane were “least preferred” by 91.7% (N = 77) participants. More than half reported head down as being their least preferred head position. There were no associations between age or gender and either most- or least-preferred head position.

**Table 1:** Most and least preferred head positions

**Head positions**	**Most preferred:**		**Least preferred:**	
	**Frequency (N = 84)**	**Percent**	**Frequency (N = 84)**	**Percent**
Centre	29	34.5	1	1.2
Centre or Down	1	1.2	0	0
Centre or Left	3	3.6	0	0
Centre or Up	2	2.4	0	0
Down	7	8.3	46	54.8
Left	9	10.7	2	2.4
Left or Right	4	4.8	2	2.4
Right	14	16.7	2	2.4
Right or Up	1	1.2	0	0
Up	13	15.5	31	36.9
Up or Left	1	1.2	0	0

### Penetration-aspiration scale (PAS) scores

In the milk condition, no associations were found between head position and PAS scores overall, for head position and gender, or for head position and age groups. There was only one episode of aspiration (with a score of 8 on the PA scale in the up head position) in the milk condition in the younger age group. The woman seemed unaware of the event, and when the swallow was repeated, she scored a PAS of 1 with no residue. This repeat swallow was not included in our analysis.

In the applesauce condition, there were no associations found between head position and PAS scores across the age groups, or for head position between genders. There were no episodes of aspiration of applesauce, but five participants (4 males and 1 female, all from the younger age group) had episodes of penetration (i.e., a score of 2 on the PA scale). One participant had an episode of penetration for the left head position, one had an episode for the center head position, and one had an episode for the up head position. One participant had an episode for the left and for the right position, while the final participant had episodes for both the right and up positions.

### Residue scores

No significant associations emerged between residue and gender (see Table 2). There was, however, a significant association between residue score and age group for the applesauce condition for the head down position: 14.8% of older participants had abnormal residue scores compared to 0% of the younger individuals (X^2^(1, N = 84) = 8.87, p = 0.003). There was also a significant association between residue and age group for the applesauce condition in the center position (X^2^(1, N = 84) = 4.33, p = 0.04), as well as for the left head position (X^2^(1, N = 84) = 4.33, p = 0.04). For each of these positions, 7.4% of older participants had abnormal residue scores compared to 0% of younger individuals. Overall there was a trend towards an association between abnormal residue with puree and age greater than 40 regardless of gender or head position (X^2^(1, N = 84) =3.74, p = .053). No other significant associations emerged.

**Table 2:** Prevalence of abnormal residue by condition

**Condition**	**Position**	**All subjects**	**Males**	**Females**	**Age < 40**	**Age ≥ 40**
Milk	Centre	0%	0%	0%	0%	0%
	Left	0%	0%	0%	0%	0%
	Right	1.2%	3.1%	0%	0%	3.7%
	Up	1.2%	0%	1.9%	1.8%	0%
	Down	0%	0%	0%	0%	0%
	All positions	2.4%	3.1%	1.9%	1.8%	3.7%
Applesauce	Centre	2.4%	6.3%	0%	0%	7.4% (p = 0.04)
	Left	2.4%	3.1%	1.9%	0%	7.4% (p = 0.04)
	Right	1.2%	3.1%	0%	0%	3.7%
	Up	7.1%	12.5%	3.8%	5.3%	11.1%
	Down	4.8%	9.4%	1.9%	0%	14.8% (p = 0.003)
	All positions	9.5%	15.6%	5.8%	5.3%	18.5% (p = 0.05)

Prevalence of abnormal residue position, gender and age.

A final exploratory analysis of residue scores stratified by age group and gender was performed (see Table 3). The significant age group effects mentioned above were particularly apparent for males in the older age group, especially for applesauce for the center position (X^2^(1, N = 52) = 4.69, p = .03), and for applesauce for the down position (X^2^(1, N = 52) = 7.28, p = .007). No males in the younger age group had abnormal residue scores for applesauce for center or down. In contrast, 20% (n = 2) of older males had abnormal residue scores for applesauce for center and 30% (n = 3) had abnormal scores for applesauce for the down position. The age group effect for the left head position did not remain significant when gender was included as one of the stratification variables.

**Table 3:** Prevalence of abnormal residue with puree

**Head position**	**Abnormal pharyngeal residue with puree**			
	**Male**		**Female**	
	**< Age 40**	**≥ Age 40**	**< Age 40**	**≥ Age 40**
Centre	0%	20% (p = 0.03)	0%	0%
Left	0%	10%	0%	5.9%
Right	0%	10%	0%	0%
Up	9.1%	20%	2.9%	5.9%
Down	0%	30% (p = 0.007)	0%	5.9%
Any Position	9.1%	30%	2.9%	11.8%

Prevalence of abnormal residue position, gender and age.

### Residue scores and preferred head position

A significant association was found for residue scores in the applesauce condition with the head up position (X^2^_corrected_(4) = 11.84, p < 0.05). It was found that 17.6% of participants who preferred left, 15.8% of those who preferred right, and 12.5% of those who preferred down had abnormal residue scores in the head up swallowing position. Those participants who preferred center or up all had normal residue scores for this position. No other significant associations emerged.

## Discussion

The primary purpose of this study was to determine whether head position was associated with differences in measures of swallowing function that would be detectable with FEES, especially differences that might explain the previously reported clinical findings. In over 300 participants, teaching the five head positions had previously resulted in improved ease of pill swallowing. Employing FEES methodology allowed us to better understand the physiological basis for these observations. The secondary goal of our research was to determine whether people without swallowing problems would have a normal FEES examination as reflected by the absence of pharyngeal residue, penetration and aspiration. We observed occasional episodes of pharyngeal residue and minor penetration of liquids above the vocal folds that is sensed and expelled even in some normal subjects. This study, however, failed to demonstrate detectable differences in PAS scores or residue for the five head positions, which is reassuring because it suggests that head-position training, at least in normal subjects, is not harmful. There was some evidence of position-sensitive effects as a function of age particularly pharyngeal residue with purees in subjects older than 40 years. In addition, there was a third, unexpected, association between residue scores and non-preferred position, in that a significant proportion of those who preferred left, right, or down had abnormal residue scores in the head up position. In contrast, the people who preferred center or up all had normal residue scores for that position. These findings suggest a tendency for people to prefer positions associated with lesser residue.

Interestingly, 14.8% of older participants had residue in the head down position that is often taught to patients with severe dysphagia and inadequate airway protection. The purpose of employing that method is to anatomically position the larynx under the tongue base to minimize the risk of aspiration, which is more likely to occur when swallowing in other positions. However, the use of head down for patients in that category does not mean that it is a comfortable or efficient position, or that people with pathology would prefer it.

One of the study’s limitations involves the FEES methodology itself, as it is not possible with FEES to observe or measure the oral phase of swallowing, nor some specific pharyngeal phase swallowing dynamics such as transit time, laryngeal elevation and cricopharyngeal relaxation. These specific aspects of swallowing are quantifiable with modified barium swallow (MBS) techniques; however the end products of dysphagia (residue, penetration, aspiration) are equally well evaluated using either procedure [10]. Subtler abnormalities of swallowing may have been detectable by MBS, but it is challenging to access MBS for research purposes due to radiation exposure associated with MBS. Another design limitation of the current study was a small proportion of participants were in the older age group, particularly age greater than 65 years. One future research project might be to examine the effects of head position on swallowing dynamics in those who are struggling to learn to swallow pills, especially in childhood. However, if this were to be done, techniques other than FEES should be explored, particularly ones that would permit quantitative measurement of swallowing dynamics.

This study has several strengths. Standardized training for head position was employed, requiring two weeks of practice for all participants. The FEES procedure was standardized. One of two otolaryngologists and one Speech Language Pathologist, each in practice almost 20 years, scored all swallows by consensus. The study was an objective evaluation of the effects of head position on swallowing not previously studied or reported. The methodology permitted an examination of both subjective (preferred position) and objective (residue and PAS scores) information, both of which were associated with subsets of participants. Finally, the sample included a broad age range and both genders.

In conclusion, our study shows that a normal FEES examination can be interpreted and reported in an office setting with confidence. Pharyngeal residue is associated with swallowing in the head up position. The incidence of pharyngeal residue may increase in middle aged and older adults. Laryngeal penetration and aspiration were not related to head position. These data provide support for the safety of head position training in normal adults who are using the method to improve their pill swallowing.

## Abbreviations

FEES: Fiber-optic endoscopic evaluation of swallowing; PAS: Penetration – Aspiration Scale (Rosenbek).

## Competing interests

The authors declare they have no conflict of interests.

## Authors’ contributions

LAB: study design, subject recruitment and training, data collection. TWM: study design, subject evaluation and data collection, manuscript preparation. AM: subject evaluation and data collection. JCD: study design, subject evaluation and data collection, data analysis. KW: subject recruitment and training, data collection. RK: subject recruitment and training, data collection. SC: data analysis. BJK: study design, draft manuscript, data analysis. All authors read, critiqued and approved the manuscript.
